# Association of vitamin D genetic pathway with asthma susceptibility in the Kurdish population

**DOI:** 10.1002/jcla.23039

**Published:** 2019-09-20

**Authors:** Rasoul Nasiri‐Kalmarzi, Mohammad Abdi, Javad Hosseini, Somayeh Tavana, Aram Mokarizadeh, Rezgar Rahbari

**Affiliations:** ^1^ Lung Diseases and Allergy Research Center Research Institute for Health Development Kurdistan University of Medical Sciences Sanandaj Iran; ^2^ Department of Pediatrics Faculty of Medicine Kurdistan University of Medical Sciences Sanandaj Iran; ^3^ Cellular and Molecular Research Center Research Institute for Health Development Kurdistan University of Medical Sciences Sanandaj Iran; ^4^ Department of Clinical Biochemistry Faculty of Medicine Kurdistan University of Medical Sciences Sanandaj Iran; ^5^ Department of Research and Development Asia Jivan Teb Science‐based Company Sanandaj Iran

**Keywords:** 1,25‐dihydroxyvitamin D3, VDBP rs7041, VDR rs2228570, vitamin D receptor, vitamin D‐binding protein

## Abstract

**Background:**

Vitamin D (Vit D) function in asthma progression has been studied well. The effects of genetic variations in Vit D pathway molecules have been also studied, although the results are contradicted. In the present study, for the first time we examined the Vit D pathway molecules included serum Vit D and vitamin D‐binding protein (VDBP) and also genetic variations in the vitamin D receptor (*VDR*) and *VDBP* in a Kurdish population with asthma.

**Methods:**

An enzyme‐linked immunosorbent assay (ELISA) method was used to measure the serum Vit D and VDBP. *VDR* rs1544410 and rs2228570 and *VDBP* rs7041 were assessed by polymerase chain reaction‐restriction fragment length polymorphism (PCR‐RFLP).

**Results:**

The serum level of Vit D significantly decreased in asthmatic patients versus controls (16.26 ± 6.76 *vs* 23.05 ± 10.57 ng/mL, *P* value = .001). We observed an indirect correlation between Vit D and clinical findings. We also found an increased level of serum VDBP in patients as compared to the controls (1044.6 ± 310.82 *vs* 545.95 ± 121.73 µg/mL, *P* value < .0001). Besides, the risk of asthma progression was increased in patients with the *VDR* rs2228570 CC and *VDBP* rs7041 GG genotypes (OR = 3.56, *P* = .0382 and OR = 2.58, *P* = .01, respectively).

**Conclusion:**

In summary, our results explain the influence of the genetic variations in *VDR* and *VDBP* in addition to Vit D and VDBP serum concentrations on asthma susceptibility in the Kurdish population.

## 1 INTRODUCTION

Asthma is a chronic inflammation of lungs that characterized by obstruction and hyper‐responsiveness of airways and causes clinical symptoms including shortness of breath, chest tightness or pain, and coughing during night or early in the morning.^1^ In addition to environmental factor effects, it has been also proved that genetic background affected the pathogenesis of asthma.^2^ Previous studies showed that a decrease in 1,25‐dihydroxycholecalciferol (1,25‐dihydroxyvitamin D3, Vit D) concentration progresses asthma symptoms due to the immune‐modulatory effects of Vit D.^3^, ^4^, ^5^ It has been previously revealed that the anti‐asthmatic effect of Vit D was applied via inhibiting the T‐cell IL4 production and suppression of Th2 immune response.^5^, ^6^, ^7^, ^8^ Besides, decreased plasma levels of Vit D result in severe asthma attacks and it has been demonstrated that Vit D supplement prevents the attacks and improves the symptoms.^9^

The correct function of Vit D is applied via proper transport in circulation by vitamin D‐binding protein (VDBP) and interaction with vitamin D response elements (VDRE) on DNA by making a triple complex with vitamin D receptor (VDR). The protective role of Vit D against asthma comes from the hypothesis that VDR over‐expresses in immune system cells especially in antigen‐presenting cells (APCs) and activated T cells.^10^ Genetic polymorphisms in the *VDR* gene effect on Vit D bind to the receptor and thus can reduce its function. Among all reported single nucleotide polymorphisms (SNPs), the association of the rs2228570 (FokI) and rs1544410 (BsmI) with the development of allergic diseases has been well studied.^11^ Both of those SNPs are located in *VDR* gene promoter and affect the VDR protein function. Despite the large numbers of studies, previous data showed the inconsistent results about the role of *VDR* SNPs in the progression of asthma in different populations.^12^, ^13^, ^14^, ^15^, ^16^, ^17^, ^18^

In addition to VDR, VDBP is bind to Vit D and has an important role in the plasma transportation of Vit D metabolites. The *GC* gene encoded VDBP and one of the most polymorphic sites in this gene is rs7041, which is located in codon 420 and is responsible for reducing the biological function of VDBP. The role of rs7041 SNP on progression of some respiratory diseases has been previously studied 19; however, there is a paucity of evidence with regard to the relation of those polymorphism with the development of asthma.^19^, ^20^, ^21^

As it was mentioned, the potential involvement of VDR in the progression of asthma has been previously investigated, although the results are controversy. Clinical heterogeneity and criteria for the selection of studied subjects are among the most important reasons in creating contradictory results. Besides, the potential role of *VDBP* rs7041 on asthma susceptibility is not well understood. Our previous study clearly revealed that alteration in Vit D metabolism including decline serum Vit D concentration, genetic variations in *VDR* and *VDBP* genes, and higher levels of serum VDBP increases the risk of chronic urticaria.^22^ Therefore, the current study was conducted to evaluate the possible association between *VDR* BsmI, FokI, *VDBP* HaeIII SNPs, and also Vit D and VDBP concentrations with asthma susceptibility in a Kurdish population.

## 2 MATERIAL AND METHODS

### 2.1 Participants

From April 2017 to August 2018, a total of 110 consecutive asthmatic patients who were admitted to the Kurdistan Asthma and Allergy Clinic and also 110 age‐ and sex‐matched healthy subjects were included in this study. The Global Initiative for Asthma (GINA) guidelines were used for the diagnosis of asthma in patients and confirmed by spirometry.^23^ We measured the forced expiratory volume in 1 s (FEV1) (% of predicted), the forced vital capacity (FVC), and FEV1/FVC ratio,^24^ and the best values were chosen for further analysis. Based on the severity of symptoms, we then classified the patients into mild, moderate, and severe persistent clusters. The control group selected from the people who came to a clinical laboratory for routine checkup after answer to a checklist and became sure that they did not have any health problem such as allergic disease, immunologic disorders, rheumatologic diseases, and history of chronic or recently acute infection. The patients were allergic asthmatics without any exposure to cigarette and without any history of other forms of allergy. All individuals were from Kurd ethnicity. All participants in the study provided their written consent prior to the study, and the study protocol was approved by the ethical committee of Kurdistan University of Medical Sciences.

### 2.2 Serum Vit D and VDBP level measurement

Five milliliter blood specimen was obtained from studied subjects through venipuncture. The serum was then separated and kept in −20°C pending evaluation. Two enzyme‐linked immunosorbent assay (ELISA) kits were used for the determination of the serum Vit D (catalog number 7725‐300, Monobind Inc.) and VDBP (catalog number CK‐E10771, Eastbiopharm, China) concentrations and the resulting absorbance read on a Stat Fax 2100 Microplate Reader (Awareness Technology Inc.) apparatus at 450 nm. The results were then expressed as ng/mL for Vit D and µg/mL for VDBP. The limit of detection (LOD) for Vit D and VDBP ELISA kits was 0.67 ng/mL and 5.41 µg/mL, respectively. According to the manufacturer's data sheets, the within‐ and between‐assay precisions were 4.95%, 5.63% and <10%, <12%, for Vit D and VDBP ELISA kits, respectively. Vit D deficiency, insufficiency, and sufficiency were defined as <10, between 10 and 30, and 30‐100 ng/mL.

### 2.3 Polymorphism genotyping

Whole blood was used for DNA extraction using a DNG‐Plus DNA extraction kit (Sinaclon). Briefly, blood specimens were lysed and DNA selectively precipitated. DNA quality and quantity were evaluated electrophoretically and photometrically. Identification of SNPs in samples was performed using a PCR‐RFLP method. The PCR condition and the primers used for PCR were based on our previous study.^22^ The PCR products were further used for genotype analysis using RFLP method. Briefly, the product size for *VDR* rs1544410, *VDR* rs2228570, and *VDBP* rs7041 was 248 bp, 341 bp, and 809 bp, respectively. The *VDR* rs1544410 was cut by FspI restriction enzyme and produced three different DNA bands on gel electrophoresis: homozygote GG (175 + 73 bp), heterozygote GA (248 + 175+73 bp), and homozygote AA (248 bp) (Figure S1). The results for *VDR* rs2228570 and *VDBP* rs7041 were as follows (Figure S1):

*VDR* rs2228570 (BseGI): homozygote TT (289 + 59 bp), heterozygote TC (341 + 289+59 bp), and homozygote CC (341bp).

*VDBP* rs7041 (BsuRI): homozygote TT (809 bp), heterozygote TG (809 + 577+232 bp), and homozygote GG (577 + 232 bp).

### 2.4 Statistical analysis

To study the frequencies of alleles and genotypes and also to evaluate the deviation of genotype frequencies from Hardy‐Weinberg equilibrium, a chi‐square test was used. The association between studied variables with risk of disease were assessed by 2 × 2 contingency table, and the odds ratio (OR) and confidence interval (CI) were evaluated. If there was a normality distribution, the parametric tests were used and data were shown as mean ± standard deviation. The independent‐samples *t* test or Mann‐Whitney statistical test was used for studying the possible differences between studied groups. The Spearman correlation coefficient was performed to evaluate the association between two variables. *P* value <.05 was considered as statistically significant. All statistical analysis was performed by SPSS 16 software (SPSS Inc.).

## 3 RESULTS

### 3.1 Demographic and clinical characteristics

The present study included 110 asthmatic patients and 110 healthy individuals. The male/female frequencies were 49 (44.54%)/61 (55.46%) for the case group and 41 (37.27%)/69 (62.73%) for the control group (*P* = .14). The age of subjects in mean ± SD was 31.34 ± 7.81 and 28.73 ± 6.89 years, for the patient and normal groups, respectively (*P* = .203). No significant differences were found between groups for sex and age of subjects. The pulmonary function characteristics were measured, and the corresponding values were as follows: FEV1 (%predicted) = 70.04 ± 20.86, FVC = 69.32 ± 19.31 and FEV1/FVC = 71.99 ± 4.75. With regard to asthma severity, 35 patients (36.8%) had mild symptoms, 24 patients (25.3%) showed moderate symptoms, and 36 patients (37.9%) had severe symptoms.

### 3.2 Measurement of Vit D and VDBP in serum samples

**Figure 1:**
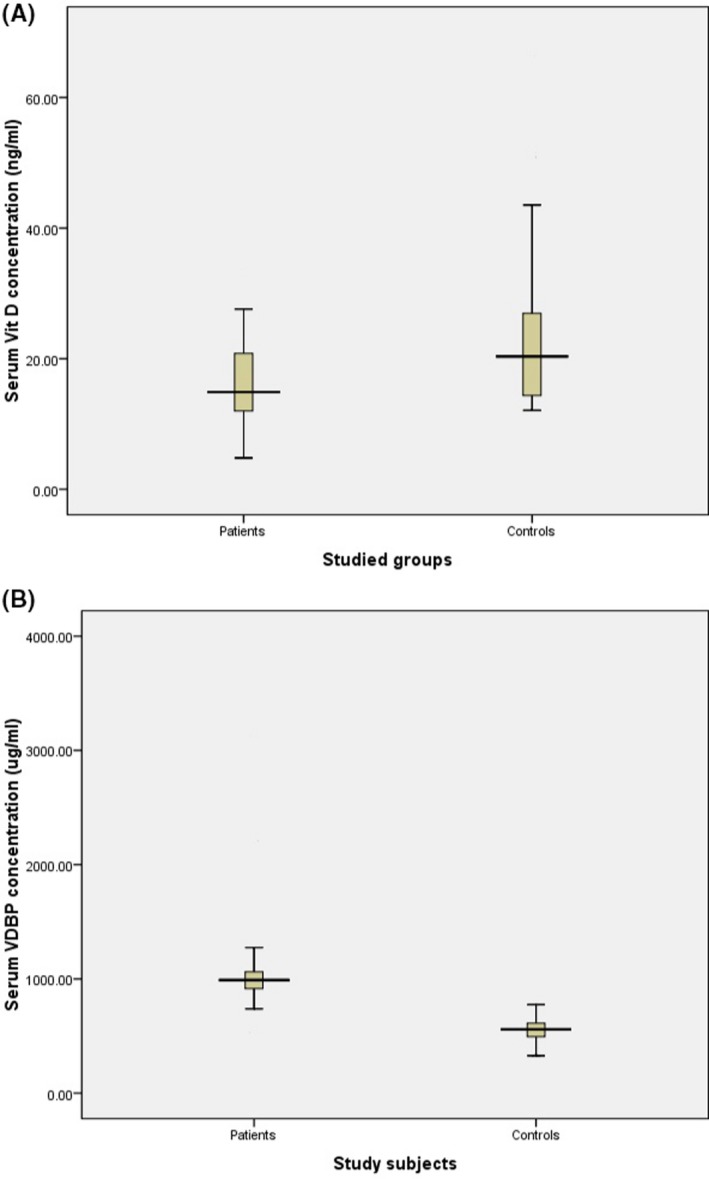
Concentration of Vit D and VDBP in studied subjects. 1A, Vit D is decreased in asthmatic patients compared to controls (*P* = .001). 1B, VDBP is significantly increased in asthmatic patients compared to controls (*P* < .0001)

Concentrations of Vit D in the patient and control groups were 16.26 ± 6.76 and 23.05 ± 10.57 ng/mL, respectively. Figure 1A shows that the serum Vit D significantly decreased in asthmatic patients compared to healthy subjects (*P* = .001). Vitamin D deficiency or insufficiency was presented in 71.42% of patients, while the corresponding value for the healthy group was 40.32% (*P* < .0001). The results indicated that decline in Vit D is a risk factor for the progression of asthma (OR = 3.04, 95% CI = 1.32‐7.00, *Z* statistic = 2.6, *P* = .0092). Differences of serum Vit D concentration between males and females were not statistically significant, although female subjects had a bit lower Vit D content (*P* = .19; 21.81 ± 11.4 *vs* 19.12 ± 8.27 ng/mL). Furthermore, our data did not show a significant association between age of subjects with serum Vit D level (*P* = .36). On the other hand, serum VDBP concentration increased in patients compared to controls (1044.6 ± 310.82 and 545.95 ± 121.73 µg/mL, respectively), and the difference between groups for VDBP was statistically significant (*P* < .0001; Figure 1B). Our results also revealed a direct correlation between high level of serum VDBP and development of asthma (Spearman's ρ = 0.61, *P* = .001).

**Figure 2:**
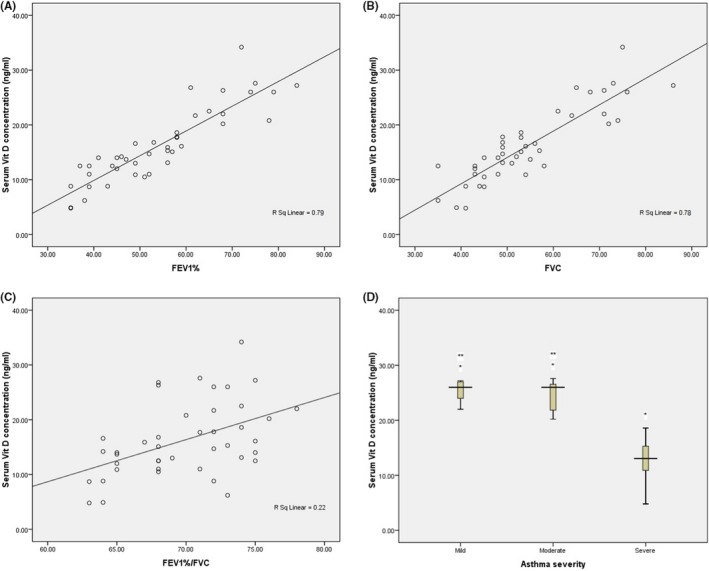
Correlation of serum Vit D with clinical findings of studied subjects. 2A, Positive correlation between serum vitamin D levels and percent predicted forced expiratory volume in 1 s (FEV1%) (*r* ^2^ = .79, *P* < .0001). 2B, Effect of serum levels of vitamin D on the forced vital capacity (FVC) was also significant (*r* ^2^ = .78, *P* < .0001). 2C, Correlation coefficient between serum vitamin D and FEV1%/FVC among patients with asthma (*r* ^2^ = 0.22, *P* = .001). 2D, Concentration of serum vitamin D was statistically significant between patients with mild (25.24 ± 2.21 ng/mL) symptoms compared to severe (12.72 ± 3.66 ng/mL) symptoms (*P < .0001). Patients with moderate (24.92 ± 4.06 ng/mL) symptoms also showed a significant difference for serum vitamin D concentration when compared to patients with severe symptoms (*P* < .0001). No significant difference was observed between mild and moderate groups for serum vitamin D concentration (**P = .8)

Linear regression analysis showed that rise in serum Vit D directly linked to higher FEV1% in asthmatic patients (*r* ^2^ = .79, *P* < .0001; Figure 2A). Our results also showed a direct correlation between Vit D with FVC (*r* ^2^ = .78, *P* < .0001) and Vit D with FEV1%/FVC (*r* ^2^ = .22, *P* = .001; Figure 2B,C). Furthermore, determination of serum Vit D based on severity of asthma showed a significant decline of Vit D in cases with severe symptoms compared to mild and moderate symptom groups (Figure 2D). In addition, no significant correlation was found between serum VDBP with clinical outcomes including FEV1%, FVC, FEV1%/FVC, asthma severity, and also the age of studied subjects (data not shown).

### 3.3 Genotype analysis

The distribution of genotype and allele frequencies for *VDR* rs1544410, *VDR* rs2228570, and *VDBP* rs7041 SNPs in patients with the asthma and control groups is shown in Table 1. All of the studied SNPs including *VDR* rs1544410, *VDR* rs2228570, and *VDBP* rs7041 were consistent with Hardy‐Weinberg equilibrium (*P* > .05).

**Table 1:** Genotypes and alleles frequencies in the patient and control groups

Genotype frequencies		Asthmatic patients (N = 110)	Controls (N = 110)	*P* Value	OR (95% CI)	Allele frequencies (%)		Asthmatic patients (%)	Controls (%)	*P* Value	OR (95% CI)
VDR rs1544410	GG	65 (59.09%)	59 (53.64%)	.2	Reference	VDR rs1544410	G	74.55	73.18	.81	0.90 (0.48‐1.7)
	GA	34 (30.91%)	43 (39.09%)	.2	0.72 (0.41‐1.27)						
	AA	11 (10%)	8 (7.87%)	.6	1.25 (0.47‐3.31)		A	25.45	26.82	.81	
VDR rs2228570	TT	51 (46.36%)	66 (60%)	.04	Reference	VDR rs2228570	T	68.18	78.18	<.05	1.67 (0.89‐3.14)
	TC	48 (43.64%)	40 (36.36%)	.27	1.55 (0.89‐2.71)						
	CC	11 (10%)	4 (3.64%)	.03	3.56 (1.07‐11.83)		C	31.82	21.82	<.05	
VDBP rs7041	TT	21 (19.1%)	29 (26.36%)	.2	Reference	VDBP rs7041	T	40	52.73	.07	1.69 (0.97‐2.96)
	TG	46 (41.8%)	58 (52.73%)	.11	1.1 (0.55‐2.17)						
	GG	43 (39.1%)	23 (20.91%)	.003	2.58 (1.21‐5.5)		G	60	47.27	.07	

**Figure 3:**
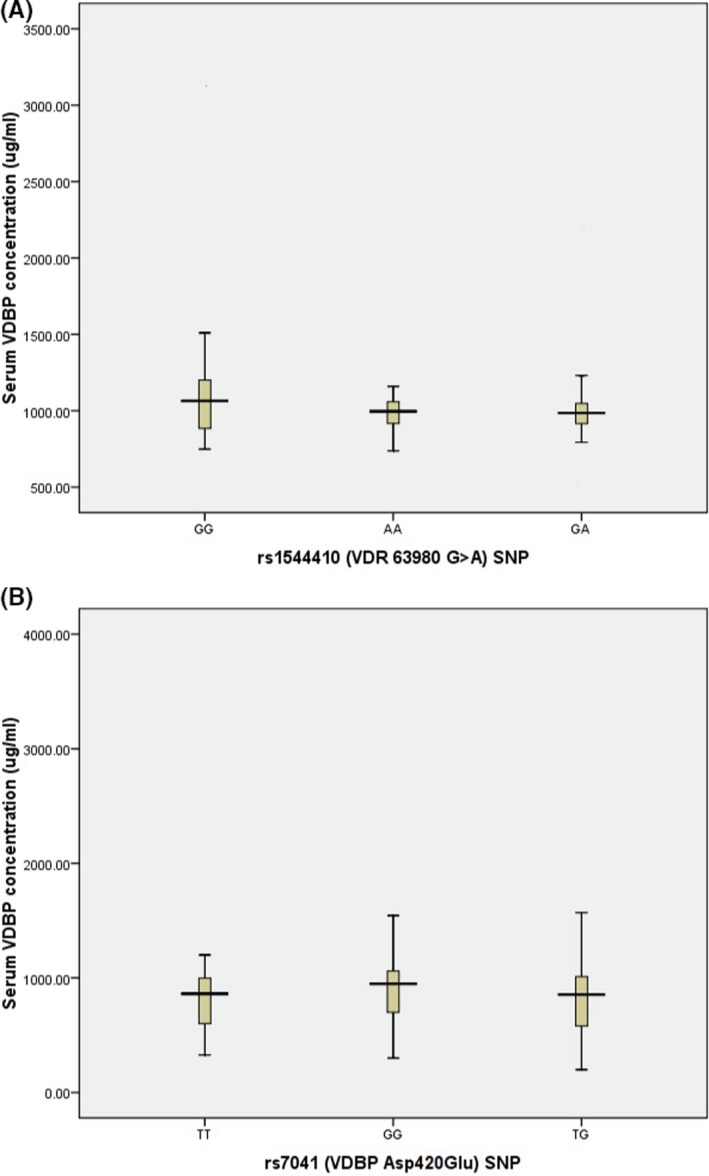
Serum VDBP concentration in different genotypes: 3A, VDBP level (in μg/mL unit) starting with the VDR rs1544410 AA genotype (989.96 ± 130.23 µg/mL) and increasing over about 1026.4 ± 229.82 µg/mL (nearly similar value) for the GA genotype up to about 1269.2 ± 737.67 µg/mL for the GG group with acceptable significance between GG genotype with AA and GA genotypes (*P* = .03). 3B, VDBP level (in μg/mL unit) was the lowest in the VDBP TG rs7041 (794.58 ± 226.99 µg/ml) and followed by the TT genotype about 816.41 ± 274.03 µg/mL (nearly similar value) and reached to maximum level in GG genotype to 974.79 ± 471.62 µg/mL, and a one‐way ANOVA analysis showed that there is a significant difference between GG genotype with TT and TG genotypes for serum VDBP level (*P* = .02)

The *VDR* rs1544410 GG genotype was more frequent in patients compared to control, although the difference was not significant (*P* = .21). Similar results were seen for GA (*P* = .21) and AA (*P* = .58) genotypes. The frequency of G and A alleles had no significant differences between patient and healthy subjects (*P* = .81), although the frequency of G allele was significantly higher in both groups versus the A allele (*P* < .0001). The clinical properties of studied subjects did not show any significant difference for *VDR* rs1544410 SNP. However, serum concentration of VDBP has increased in GG genotype compared to heterozygote GA and homozygote AA genotypes (1269.2 ± 737.67, 1026.4 ± 229.82, and 989.96 ± 130.23 µg/mL, respectively) (*P* = .03; Figure 3A). Similar results were seen for serum VDBP in patients with GG genotype compared to the AA + GA group (*P* = .001, Table S1).

Genotype analysis of *VDR* rs2228570 showed the higher prevalence for TT genotype in both cases and controls compared to CC genotype. The TT genotype was also higher in control subjects compared to patients with asthma (*P* = .0424), while the CC genotype had significantly higher frequency in patients as compared to controls (*P* = .0307). Similar significant differences were also observed when compared to two groups based on T and C alleles (*P* < .05). Our data showed that CC genotype has a potential role in the progression of asthma (OR = 3.56, 95% CI = 1.07 to 11.83, *P* = .0383). However, we observed that *VDR* rs2228570 is not a risk factor for asthma development in allele level (OR = 1.67, 95% CI = 0.89 to 3.14, *P* = .11). Furthermore, analysis of studied groups did not show any correlation between *VDR* rs2228570 SNP and clinical findings of asthmatic patients.

The rate of the GG‐rs7041 was increased in the patient group compared to healthy subjects (39.1% vs 20.91%, *P* = .003). In addition, GG genotype was a potential risk factor for asthma development (OR = 2.58, 95% CI = 1.21‐5.5, *P* = .014). Our data showed that *VDBP*‐rs7041 has no significant association with clinical findings included serum Vit D, FEV1%, FVC, and FEV1/FVC. However, serum VDBP concentration was observed to be increased in GG genotype compared to TT and TG genotype (974.79 ± 471.62, 816.41 ± 274.03, and 794.58 ± 226.99 µg/mL, respectively) (*P* = .02, Figure 3B). In addition, the VDBP serum concentration was found to be increased in patients with GG genotype when compared to the TT + TG group (*P* = .028, Table S1). We also found that the GG‐rs7041 was a potential risk factor for asthma development compared to TT + TG (OR = 2.43, 95% CI = 1.33‐4.42, *P* = .004; Table S2).

## 4 DISCUSSION

Here, we demonstrated that serum Vit D level declined in patients with asthma. We also observed the higher concentration of VDBP in those patients. A significant indirect correlation was found between serum Vit D and VDBP. Decline in Vit D levels linked to clinical findings included FEV1%, FVC, FEV/FVC, and asthma severity, while increased levels of the serum VDBP did not link with those characteristics. We also evaluated the frequency of *VDR* rs1544410, rs2228570, and *VDBP* rs7041 SNPs and the progressive role of these variations in asthma patients. The CC genotype of the *VDR* rs2228570 and the GG genotype of *VDBP* rs7041 were observed to be significantly increased in patients compared to controls and this increase associated with asthma development. To the best of our knowledge, our study is the first one that concurrently assessed the role of Vit D pathway including VDR and VDBP in the progression of asthma in the Kurd ethnicity.

The effect of Vit D on reducing the asthma exacerbation has been previously well studied. Vit D concentration inversely correlates with asthma progression and pulmonary function. Furthermore, Vit D supplements showed a protective role among patients with asthma.^25^ The immune‐modulatory effects of Vit D applies through direct targeting of immune system cells included B cells, T cells, dendritic cells, and macrophages.^26^, ^27^ Similarly, our data indicated decrease of Vit D in patients and we also observed a direct correlation between Vit D and clinical findings in the studied subjects.

It has been previously showed that higher level of VDBP decreases the bioactivity of Vit D. Gupta et al 28 showed that VDBP increased in biological fluids of children with severe therapy‐resistant asthma and the association between rises in BAL‐VDBP with asthma control was significant. More recently, Jiang et al 29 revealed that VDBP increased in patients with steroid‐resistant asthma as compared to steroid‐sensitive asthma. They proposed that serum VDBP may apply as a useful biomarker for predicting the resistance to steroid in asthma patients. In line with these studies, we found that serum VDBP was increasing in asthma patients, although our results did not show any link between VDBP and clinical characteristics.

It has been shown that variations in *VDR* gene may impact on progression of asthma. Tizaoui et al 30 studied a total of eight previous case‐control studies and showed a significant association between homozygous wild type of rs1544410 with risk of asthma (OR = 2.017, 95% CI = 1.236‐3.851, *P* = .017), although it seems that the *VDR* rs2228570 is not a probable risk factor for development of asthma in the codominant model (OR = 1.187, 95% CI = 0.975‐1.446, *P* = .088). They proposed that study features may influence on the association between rs2228570 and asthma susceptibility. More recently, Zhao et al 31 investigated the possible correlation between polymorphisms in *VDR* gene and susceptibility to childhood asthma. The highest significant odds ratio was seen in the case of ApaI polymorphism in homozygous and allele models (1.674 and 1.221, respectively). They were also found that Asian ethnicity with ApaI SNP has a higher risk to the progression of asthma. With regard to rs2228570 and rs1544410 polymorphisms, they showed that *VDR* rs1544410 may be significantly associated with progression of asthma in homozygous (OR = 1.462, 95% CI = 1.016‐2.105, *P* = .041) and allele level (OR = 1.181, 95% CI = 1.006‐1.386, *P* = .042) in Caucasian population. Similarly, *VDR* rs2228570 was also a risk factor for childhood asthma susceptibility in dominant (OR = 1.281, 95% CI = 1.055‐1.555, *P* = .012) and allele (OR = 1.591, 95% CI = 1.052‐2.405, *P* = .028) models in Caucasian patients. In contrast, Hou et al 32 did not find any correlation between *VDR* rs1544410 with childhood bronchial asthma. In our study, no association was found between *VDR* rs1544410 loci with asthma susceptibility in the Kurdish population. However, we found that *VDR* rs2228570 polymorphism should be considered as a potential risk factor for the progression of asthma in the Kurd ethnicity.

There was a paucity of studies that investigated the impact of *VDBP* genetic variations on asthma susceptibility. The effect of VDBP SNPs on progression of COPD has been previously investigated.^20^ Those results revealed that GC1f and GC2 alleles may be linked to sputum hypersecretion in COPD patients. In another study, Ismail et al 33 observed that homozygous GG genotype of the *VDBP* rs2282679 may be linked to asthma susceptibility and clinical findings in asthmatic patients including lung functions, asthma severity, and concentration of IgE and Vit D. In addition, Randolph et al 34 showed that carriers of C allele of the *VDBP* rs7041 had a higher risk to the progression of respiratory syncytial virus bronchiolitis and later asthma development (OR = 1.12, 95% CI = 1.02‐1.4, *P* = .03). Li et al 21 also showed that compared to GC1, patients with GC2 haplotype are more susceptible for the development of asthma. Contrarily, *VDBP* GC1s have been proposed as a protective factor for the progression of asthma when compared to GC1f/1f genotype in the Hispanic population.^35^ More recently, Fawzy et al 36 observed that GG genotype and G allele of the *VDBP* rs7041 are a potential risk factor for asthma development, while rs4588 AA genotype and A allele had a protection role in that study. In line with previous results, our study revealed a provocative effect for GG genotype of *VDBP* rs7041 in asthma development of the Kurdish population.

Our study had several limitations that must be noted; first limitation is linked to the numbers of studied SNPs. We only evaluated two *VDR* SNPs and one VDBP polymorphism, while there are more SNPs that could influence on disease progression. Second, we could not rule out the effects of environmental factors on studied subjects. Finally, we only sampled single blood specimen and subsequently measured Vit D and VDBP in those samples; hence, the role of biological variations must be considered.

In conclusion, we have identified that decrease of Vit D is associated with high level of VDBP and those phenomenon involve in the pathogenesis of asthma. Besides, we found that *VDR* rs2228570 and *VDBP* rs7041 correlated to asthma exacerbation and should be considered as potential genetic factors in asthma progression in the Kurdish population.

## CONFLICT OF INTEREST

Dr R Nasiri‐Kalmarzi declares no potential conflicts of interest with respect to the research, authorship, and/or publication of this article. Dr M Abdi has received research grants from Kurdistan University of Medical Sciences. Mr J Hosseini declares that he has no conflict of interest. Mrs S Tavana declares that she has no conflict of interest. Dr A Mokarizadeh declares that he has no conflict of interest. Mr R Rahbari declares that he has no conflict of interest.

## AUTHOR CONTRIBUTIONS

All authors contributed equally in this work.

## ETHICAL APPROVAL

All procedures performed in studies involving human participants were in accordance with the ethical standards of the ethics committee of Kurdistan University of Medical Sciences and with the 1964 Helsinki Declaration and its later amendments or comparable ethical standards.

## INFORMED CONSENT

Informed consent was obtained from all individual participants included in the study.
