# Supplementary material for: Association of vitamin D genetic pathway with asthma susceptibility in the Kurdish population
Source: J Clin Lab Anal. 2019 Sep 20;34(1):e23039. doi: 10.1002/jcla.23039 (PMC6977155; doi:10.1002/jcla.23039)
Supplement: Supplementary file 1 [file JCLA-34-e23039-s001.docx]

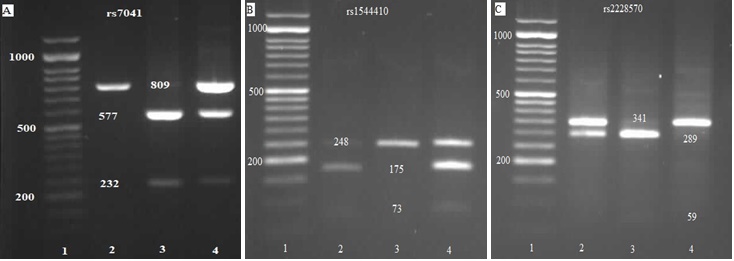


Supplement figure 1: PCR-RFLP pattern of the studied SNPs

A: VDBP rs7041, Line 1 50bp DNA ladder, Line 2 rs7041 TT, Line 3 rs7041 GG, Line 4 rs7041 TG

B: VDR rs1544410, Line 1 50bp DNA ladder, Line 2 rs1544410 GG, Line 3 rs1544410 AA, Line 4 rs1544410 GA

C: VDR rs2228570, Line 1 50bp DNA ladder, Line 2 rs2228570 TC, Line 3 rs2228570 TT, Line 4 rs2228570 CC

| Supplement table 1: Clinical and laboratory characteristics among VDBP and VDR genotypes according to different genetic models | | | | | | | | | | | | | | | | | | |
| --- | --- | --- | --- | --- | --- | --- | --- | --- | --- | --- | --- | --- | --- | --- | --- | --- | --- | --- |
|  | VDBP rs7041 | | | | | | VDR rs2228570 | | | | | | VDR rs1544410 | | | | | |
|  | TT+TG | GG | *p* value | TG+GG | TT | *p* value | TT+TC | CC | *p* value | TC+CC | TT | *p* value | GG+GA | AA | *p* value | GA+AA | GG | *p* value |
| Vit D (ng/ml) | 20.06 ± 9.75 | 21.29 ± 10.34 | 0.58 | 20.81 ± 10.75 | 19.43 ± 7.27 | 0.54 | 21.53 ± 11.04 | 19.65 ± 9.13 | 0.36 | 20.53 ± 10.2 | 19.08 ± 6.08 | 0.7 | 20.11 ± 9.8 | 21.58 ± 10.56 | 0.6 | 20.9 ± 10.13 | 15.3 ± 5.26 | 0.09 |
| VDBP (μg/mL) | 808.56 ± 257.00 | 974.79 ± 471.62 | 0.028 | 869.77 ± 377.46 | 816.41 ± 274.03 | 0.81 | 875.04 ± 336.42 | 869.45 ± 366.4 | 0.93 | 865.2 ± 359.56 | 947.03 ± 257.77 | 0.46 | 867.29 ± 373.85 | 989.96 ± 130.23 | 0.81 | 837.52 ± 295.35 | 1269.2 ± 737.67 | 0.001 |
| FEV1% | 70.03 ± 20.92 | 70.4 ± 21.27 | 0.94 | 70.37 ± 21.7 | 69.58 ± 18.96 | 0.87 | 73.17 ± 22.16 | 67.57 ± 19.64 | 0.2 | 69.34 ± 19.9 | 69.19 ± 17.93 | 0.99 | 68.53 ± 21.02 | 76.5 ± 19.44 | 0.15 | 70.36 ± 20.66 | 67.3 ± 23.55 | 0.7 |
| FVC | 70.17 ± 19.76 | 68.34 ± 19.68 | 0.67 | 69.69 ± 20.27 | 68.92 ± 18.08 | 0.87 | 72.14 ± 20.52 | 67.08 ± 18.75 | 0.21 | 70.05 ± 21.23 | 70.0 ± 18.4 | 0.49 | 67.92 ± 19.91 | 75.28 ± 17.57 | 0.15 | 69.74 ± 19.66 | 65.7 ± 19.86 | 0.55 |
| FEV1%/FVC | 71.92 ± 4.74 | 72.06 ± 4.89 | 0.89 | 71.66 ± 4.76 | 72.88 ± 4.78 | 0.28 | 72.33 ± 5.23 | 71.72 ± 4.37 | 0.53 | 72.11 ± 4.85 | 71.0 ± 3.89 | 0.71 | 71.94 ± 4.82 | 72.22 ± 4.57 | 0.82 | 71.85 ± 4.85 | 73.2 ± 3.79 | 0.39 |

| Supplement table 2: OR and 95%CI for association between VDBP rs7041, VDR rs2228570 and VDR rs1544410 SNPs and asthma risk according to different genetic models | | | | | |
| --- | --- | --- | --- | --- | --- |
| Genotype | | Asthmatic patients (n=110) | Controls (n=110) | *p value* | OR (95 % CI) |
| VDBP rs7041 | TT+TG | 67 | 87 | 0.004 | 2.43 (1.33 – 4.42) |
|  | GG | 43 | 23 |  |  |
|  | TG+GG | 89 | 81 | 0.2 | 0.66 (0.35 – 1.24) |
|  | TT | 21 | 29 |  |  |
| VDR rs2228570 | TT+TC | 99 | 106 | 0.07 | 2.94 (0.91 – 9.55) |
|  | CC | 11 | 4 |  |  |
|  | TC+CC | 59 | 44 | 0.05 | 1.74 (0.99 – 2.96) |
|  | TT | 51 | 66 |  |  |
| VDR rs1544410 | GG+GA | 99 | 102 | 0.47 | 1.42 (0.55 – 3.67) |
|  | AA | 11 | 8 |  |  |
|  | GA+AA | 45 | 51 | 0.41 | 0.80 (0.47 – 1.37) |
|  | GG | 65 | 59 |  |  |
